# Supplementary material for: The effect of intrapartum prolonged oxygen exposure on fetal metabolic status: secondary analysis from a randomized controlled trial
Source: Front Endocrinol (Lausanne). 2023 Jun 27;14:1204956. doi: 10.3389/fendo.2023.1204956 (PMC10335765; doi:10.3389/fendo.2023.1204956)
Supplement: Supplementary file 3 [file DataSheet_3.zip › Result-X101SC21092977-Z01-J001-B1-42/3.MetDiffScreening/O.vs.A/O.vs.A_pos.xls.volcano.pdf]

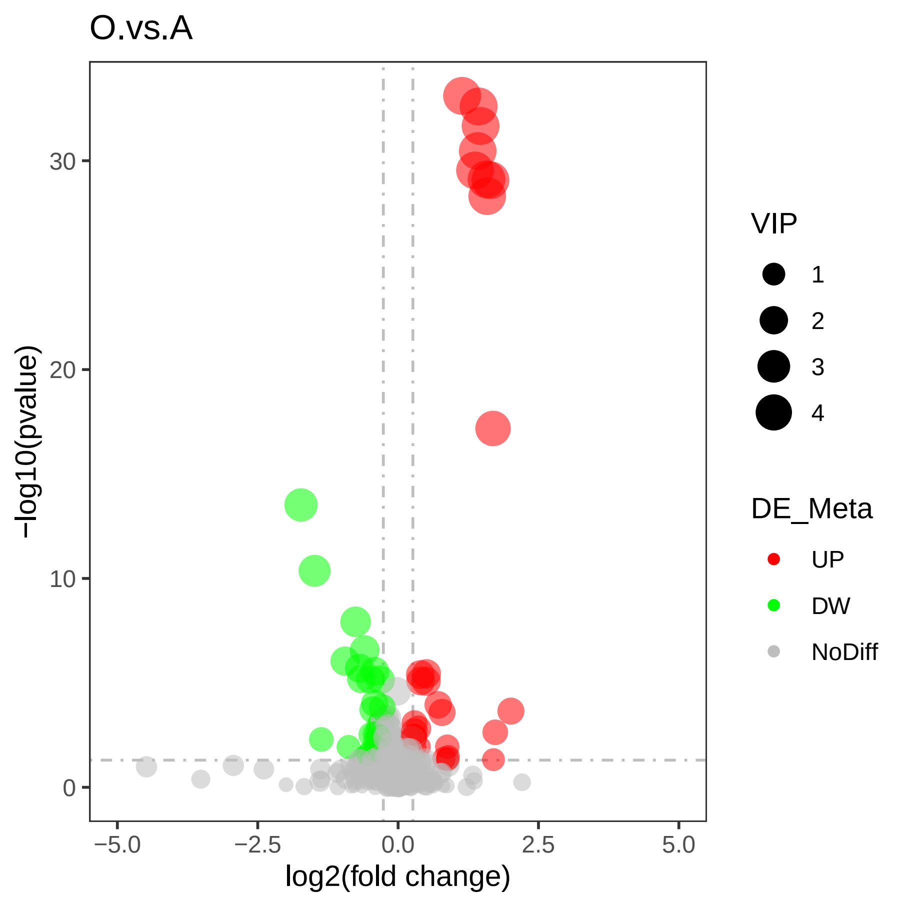

O.vs.A
30
20
10
0
−5.0
−2.5
0.0
2.5
5.0
−log10(pvalue)
log2(fold change)
VIP
1
2
3
4
DE_Meta
UP
DW
NoDiff
